# Peptide YY and neuropeptide Y in regulation of pain and spatial learning and memory

**DOI:** 10.1186/2050-6511-13-S1-A58

**Published:** 2012-09-17

**Authors:** Piyush Jain, Ahmed Hassan, Herbert Herzog, Peter Holzer

**Affiliations:** 1 Research Unit of Translational Neurogastroenterology, Institute of Experimental and Clinical Pharmacology, Medical University of Graz, 8010 Graz, Austria; 2 Neuroscience Research Program, Garvan Institute of Medical Research, Darlinghurst NSW 2010, Australia

## Background

Peptide YY (PYY) and neuropeptide Y (NPY), two members of the pancreatic polypeptide-fold family of biologically active peptides, play important roles in the regulation of food intake, energy homeostasis and emotional-affective processes. While NPY also participates in nociceptive processing and cognition, the implication of PYY in pain, learning and memory has been little studied. Therefore, male wild-type, PYY knockout (PYY(−/−)), and NPY plus PYY double knockout (NPY(−/−);PYY(−/−)) mice were studied for their sensitivity to noxious heat in the plantar test and for their spatial learning and memory performance in the Barnes maze.

## Methods

In the plantar test mice were habituated in small compartments for 1 h. Subsequently the plantar side of the right and left hind paw was exposed to an infrared source through a glass plate (cut off time: 15 s). The withdrawal response was assessed at two infrared intensities, and the withdrawal latency recorded. In the Barnes maze test, mice were placed in the middle of a circular maze with 20 holes. The task of the mice was to find a target hole with an escape box within a preset time. The animals received 4 training runs on the first day and 3 trainings daily for the following 3 days. The first probe trial was performed on day 5 and the second probe trial on day 12, to check short-term and long-term memory, respectively. Target hole visits, preference (target hole visits/total visits) and latency (time taken to reach the target hole) were measured.

## Results

In the plantar test at the higher infrared intensity, PYY(−/−) and NPY(−/−);PYY(−/−) mice presented with a significant decrease (p < 0.01 and p < 0.05) in withdrawal latency when compared with wild-type mice. At the lower infrared intensity, the withdrawal latency of PYY(−/−) mice was also significantly shorter (p < 0.01) than that measured in wild-type mice, whereas NPY(−/−);PYY(−/−) mice did not significantly differ from wild-type mice. In the Barnes maze test, significant differences were only found during probe trial 1 when NPY(−/−);PYY(−/−) mice visited the target hole less often (p < 0.01) than wild-type mice. In addition, the total number of hole visits made by NPY(−/−);PYY(−/−) mice was significantly (p < 0.01) lower than that made by wild-type animals.

## Conclusions

The most important observation of this study is that genetic deletion of the gut hormone PYY decreases the pain threshold to noxious heat. Additional knockout of the neuropeptide NPY, which is known to play a role in nociception, did not increase the hyperalgesic effect of PYY knockout. In contrast, the impact of PYY and NPY deletion on the performance in the Barnes maze was modest, and the decrease in target hole visits was probably due to reduced locomotion and did not reflect an impairment of short term memory.

